# The Legacy of Sir Ronald Ross: From Malaria Research to Multifaceted Achievements

**DOI:** 10.7759/cureus.65999

**Published:** 2024-08-02

**Authors:** Medhavi Malpe, Sonali G Choudhari, Nikhilesh Nagtode, Pramita Muntode Gharde

**Affiliations:** 1 Department of Community Medicine, Jawaharlal Nehru Medical College, School of Epidemiology and Public Health, Datta Meghe Institute of Higher Education and Research, Wardha, IND; 2 Epidemiology and Public Health, Public Health Department, Gondpipri, Chandrapur, IND

**Keywords:** mosquito, malaria, historical vignette, medical scientist, malaria mosquito cycle, malaria transmission

## Abstract

Sir Ronald Ross (13th May 1857 - 16th September 1932), a British doctor, was awarded the Nobel Prize in Physiology/Medicine in 1902 for research on the spread of malaria. This article highlights the multifaceted and significant scientific work by Ross. In 1897, he demonstrated that malaria is transmitted via mosquito bites and that malaria parasites exist in the gastrointestinal tract of the mosquito. Ross elucidated the transmission cycle in mosquitoes and birds infected with *Plasmodium*. His 25-year career in the Indian Medical Service laid the foundation for his ground-breaking work in malaria. Besides medicine, Ross excelled in poetry, music, and mathematics. The London School of Hygiene & Tropical Medicine has a frieze dedicated to 23 people chosen for their accomplishments in the field of public health, one of whom is Sir Ronald Ross. His legacy lives on through various honors and institutions, like the Ross Institute.

## Introduction and background

Sir Ronald Ross’s incredible contribution to discovering malaria parasite transmission is well known. He received the Nobel Prize in Physiology and Medicine in 1902 for his studies of malaria disease transmission. Confirmation of the presence of malarial parasites in the mosquito’s gastrointestinal tract in 1897 confirmed that mosquitoes were vectors of malaria, which paved the way for a fight against the disease [1]. Ross was a brilliant and versatile mind and was a multi-talented individual who authored poetry, released novels, and crafted music [2]. He was a beginner artist and mathematician as well. He dedicated a quarter-century to the Indian Medical Service. During his service, he made a revolutionary medical breakthrough [1]. He began his work on malaria in the year 1892. In 1894, he planned to test Laveran and Manson's hypothesis about mosquitoes' involvement in the transmission of disease in India. Despite various attempts for two and half years, Ross finally achieved the life cycle of malaria parasites in mosquitoes as a confirmation of the theory proposed by Laveran and Manson [2].

Due to his discoveries, Ronald Ross positively impacted medical science for many more years after his death. His work done on malaria is beneficial and has saved many lives and still shaping health policies and research [1]. Institutions and awards, such as the Ross Institute at the London School of Hygiene & Tropical Medicine, stand as a testament to his enduring legacy [2]. What stands out about Ronald Ross's life is his persistent pursuit of experiments in the face of numerous setbacks and with no financial or material assistance from the institution he led.

Ross was honored with the Nobel Prize for Physiology/Medicine, though not as soon as he anticipated [3]. He was honored with numerous awards and was granted Honorary Membership in scholarly societies across Europe and various other continents. In 1910, during the centennial celebration of the Caroline Institute in Stockholm, he conferred an honorary Doctor of Medicine degree. While his liveliness and focused pursuit of truth led to conflicts with certain individuals, he had a large group of peers across Europe, Asia, and America who praised him for both his character and his brilliance [2].

## Review

Sir Ronald Ross’s life and career

**Figure 1:**
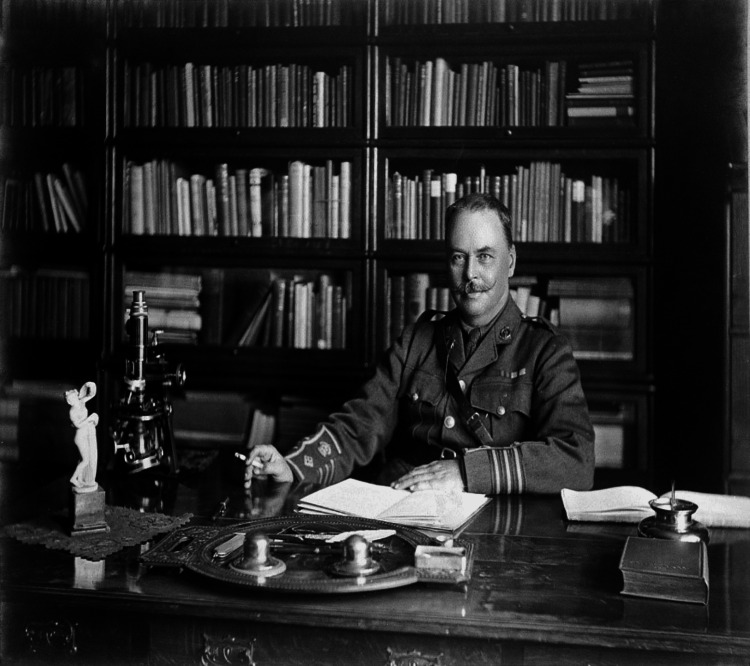
Portrait of Sir Ronald Ross at his desk. Source: Under Creative Commons Attribution CC BY 4.0 license [7]. Credit: Photograph by Elliott & Fry, Wellcome Library, London.

**Figure 2:**
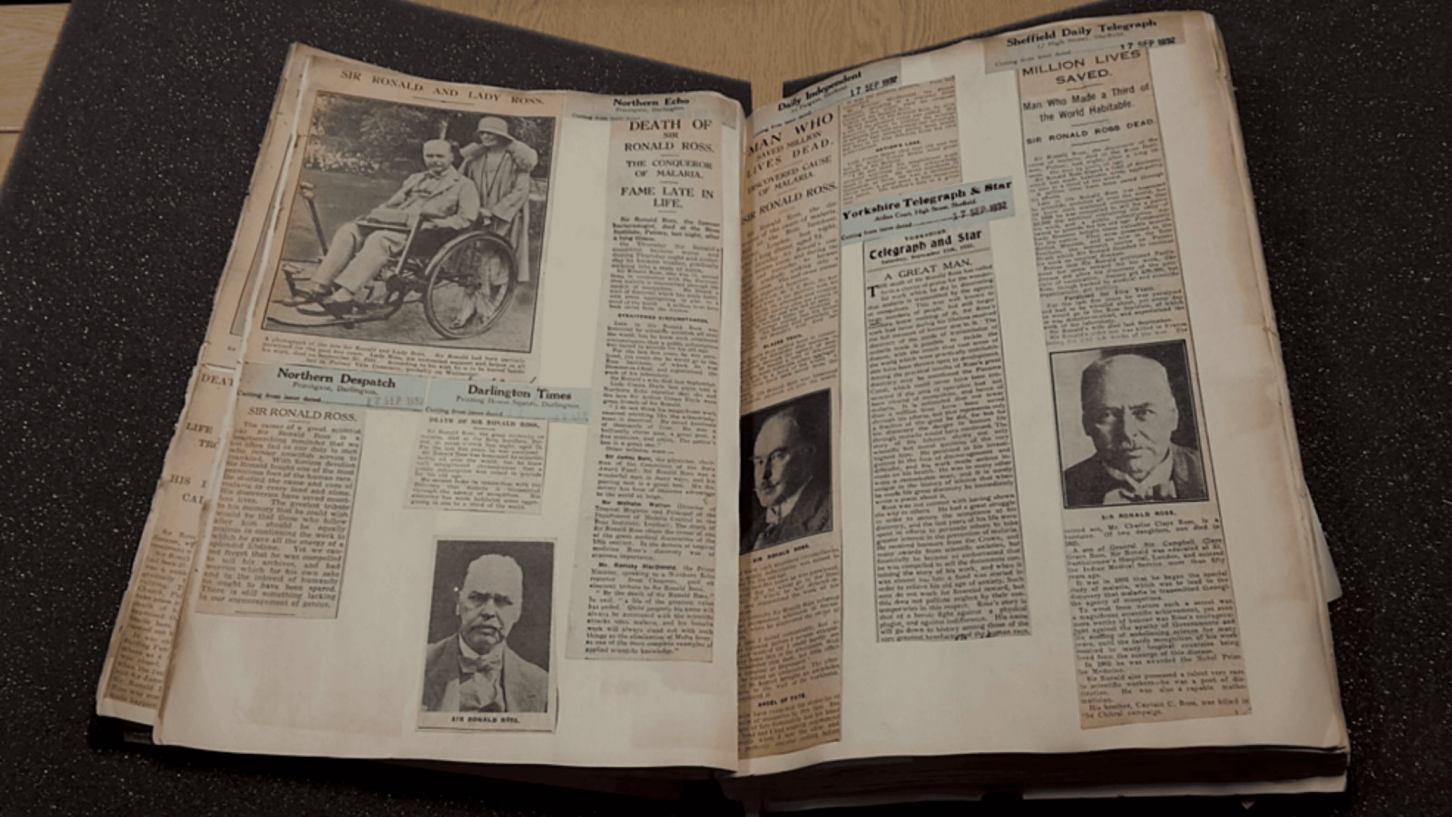
Selection of newspaper clippings from Ross. Source: Library, Archive & Open Research Services blog [8].

Sir Ronald Ross was born in India on May 13, 1857, to a Scottish Army Officer and his spouse [4]. When he was eight years old, he was relocated to reside on the Isle of Wight in England. Ross went to elementary schools in the Ryde and, in 1869, he was enrolled in a boarding school at Springhill, close to Southampton, for high school education. Since the early years of his life, he harbored a love for poetry, songs, literature, and mathematics. When he was 14 years old, Ross received an award for his mathematics skills. At age 16, in 1873, he achieved the top position in a local examination of drawing at Oxford and Cambridge [5]. Following his studies in England, he matriculated at St. Bartholomew's Hospital Medical College in 1874 [4]. After a few years, Ross joined the Liverpool School of Tropical Medicine and ultimately became a professor of Tropical Medicine at the University of Liverpool. In 1912, Ross was appointed physician for tropical illnesses at the King's College and Hospital in London, and later Director of the Ross Institute for Tropical Diseases, which was named in his honor [3]. Figure 1 depicts the portrait of Sir Ronald Ross at his desk. He married Rosa Bessie Bloxam in 1889. Ross survived his wife's death in 1931 and died a year later on September 16, 1932, at the Ross Institute in London, following a long illness [2]. Ross also developed mathematical models for the transmission of malaria. His theories lay the groundwork for epidemiology, which aids in predicting and controlling infectious disease outbreaks [6]. Figure 2 shows a selection of newspaper clippings from Ronald Ross.

The turning point: research on malaria

Ronald Ross did not conform to conventional standards or typical normative ideas within academic naturalism. He developed a fresh concept for parasitology and endorsed a theory centered around the principle that every illness must be linked to a specific biological agent (pathogen) [9]. Ross’s interest in malaria began in the early 1890s when he was stationed in India. At that time, malaria was a major health issue, but its transmission mechanism was not understood. Influenced by the work of Sir Patrick Manson, who hypothesized that mosquitos contributed to the transmission of malaria, Ross embarked on research that would eventually validate this theory [10]. On August 16, 1897, Ross allowed 10 *Anopheles* mosquitoes to feed on a malaria patient who had volunteered, named Abdul Kadir [11]. Since Ross was not an entomologist, the only entomology book he owned was intended for anglers. He classified the mosquitoes he was researching as grey or barred-back (A), brindled (B), and dappled-winged (C) [12]. Over the following days, he dissected the mosquitoes but found no malarial parasites until August 20, when he examined the stomach tissue of one and found cells with clusters of black granules resembling Laveran's parasites. The next day, he found even larger parasites in another mosquito's gut, confirming the link between mosquitoes and malaria. Ross named August 20 "Mosquito Day." Although his transfer prevented further work with humans, he demonstrated the transmission of avian malaria via mosquitoes. In 1898, Giovanni Grassi confirmed the same process with *Anopheles* mosquitoes and humans [13].

Challenges in malaria research work

**Figure 3:**
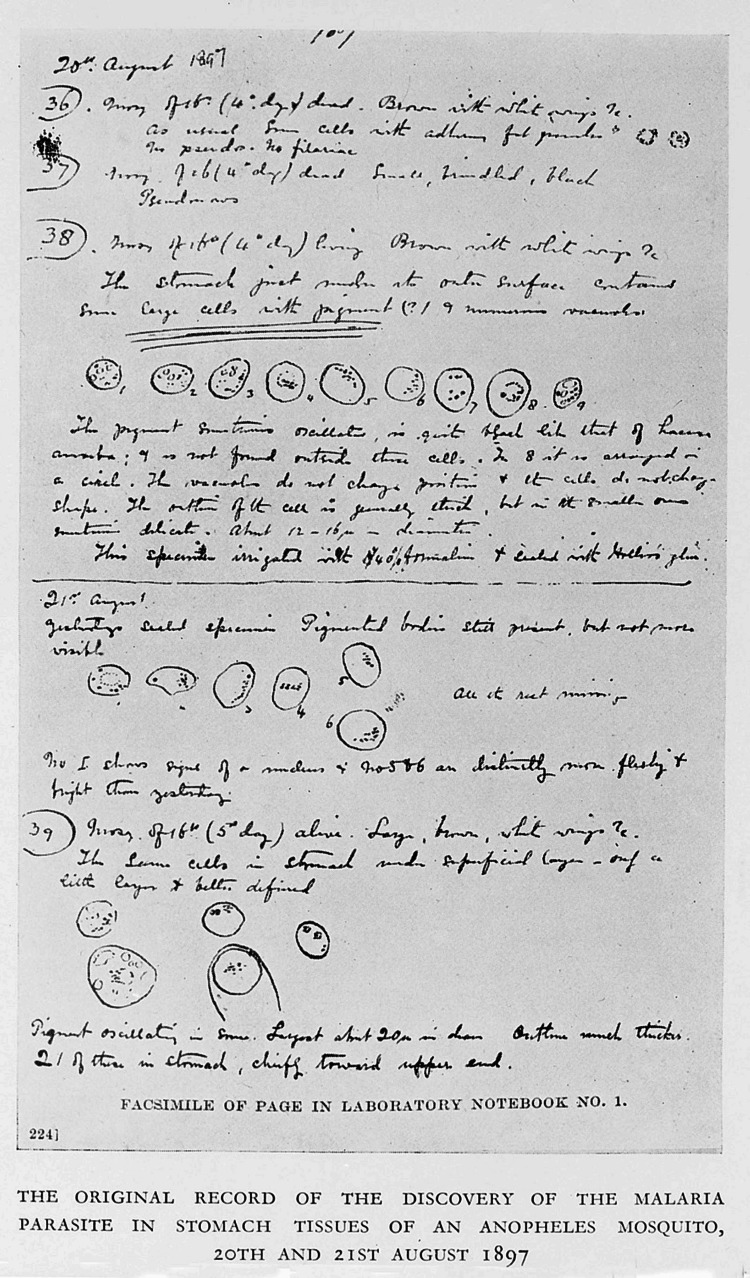
A page from Sir Ronald Ross's notebook, recording the "pigmented bodies" in mosquitoes, which he later identified as malaria parasites (20th & 21st August 1897). Source: This image is licensed under the Creative Commons Attribution 4.0 International license [14]. Credit: Photograph by Mégroz RL, Wellcome Library, London.

Ross became constantly incensed by the government's lack of support, which he referred to as "administrative barbarism" for scientists working on medical research [11]. Not every step of his journey was easy sailing. At times, he suspected that some of the responsibilities assigned to him were just to show him his place. He was once assigned to work for the Rajputana Medical Service in a little town called Kherwara. In another event, Ross asked permission from the Surgeon General to continue work on malaria but was denied due to a lack of approval from higher authorities. Because of this and considering that he had invested a significant amount of money into his research, he decided to step down from the Indian Medical Services [3]. On 22 February 1899, Ronald Ross eventually departed from India [11]. Figure 3 depicts the page from Sir Ronald Ross's diary entry describing the moment he discovered the malaria vector.

Legacy

**Figure 4:**
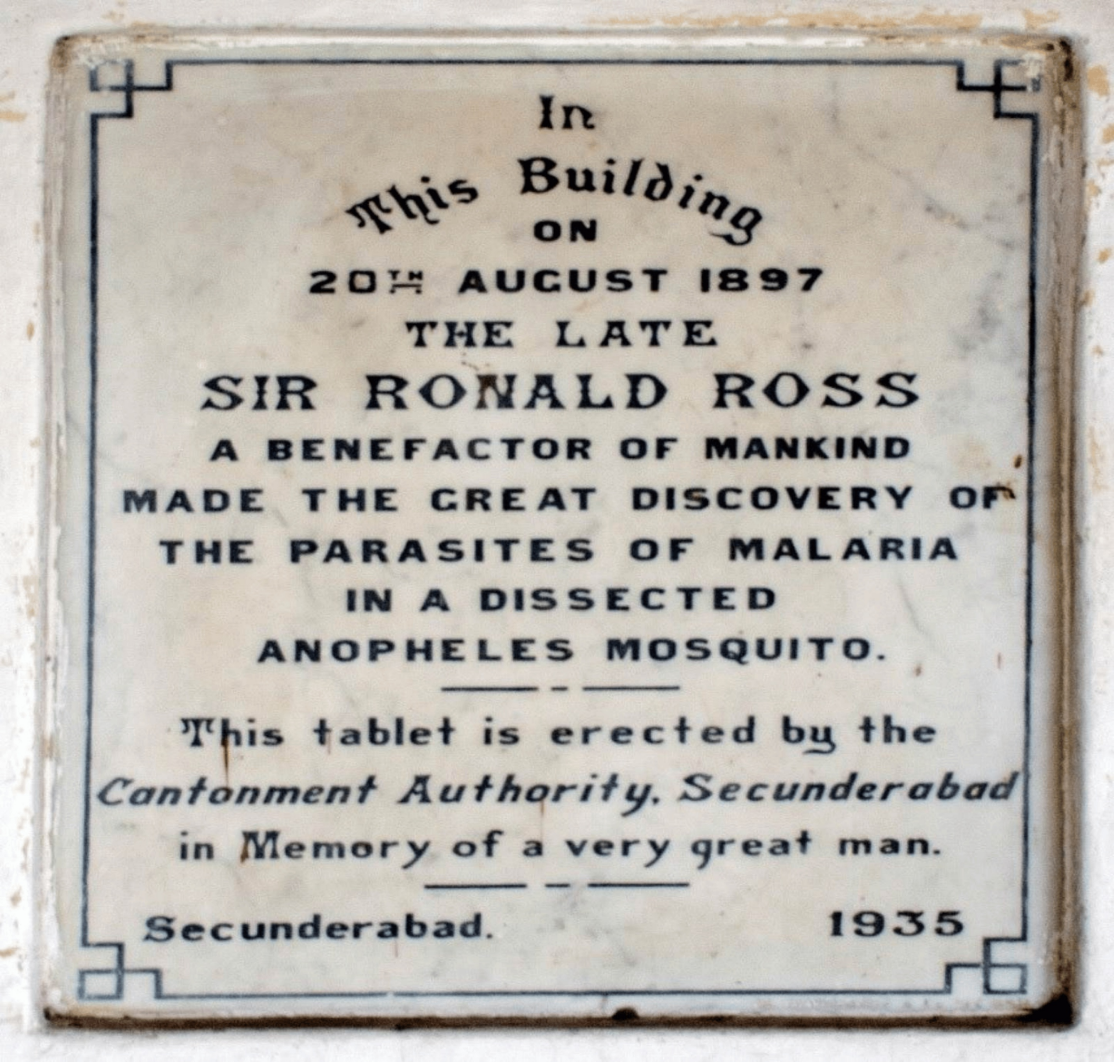
A plaque at the Sir Ronald Ross Institute of Parasitology, Begumpet, Hyderabad. Under Creative Commons Attribution-Share Alike 4.0 International license. Source: Wikimedia Commons [17].

Ross worked by leading numerous international initiatives to stop malaria-related deaths and misery. Without his contributions and the efforts of other early pioneers who put their lives in danger to learn more about what was and is still one of the worst killers in the world, none of this would have been possible [15]. The Sir Ronald Ross Institute of Parasitology is housed in the same building where Ross's futuristic finding occurred in Hyderabad. There's an arch honoring Ross on the north wall of the institute. Two marble plaques are positioned on either side of the Ross medallion, which is located in the center, directly above the arch. The poem on the right was written by Ross to commemorate his historic discovery, while the one on the left talks about the discovery [16]. Now known as Sir Ronald Ross Laboratory, it is the same facility where Ross uncovered the lifecycle of the lethal parasite. Today, it serves as a laboratory for testing for malaria. The London School of Hygiene & Tropical Medicine's frieze honors 23 pioneers who were selected for their achievements in the field of public health, including Sir Ronald Ross [2]. Figure 4 depicts a plaque at Sir Ronald Ross Institute of Parasitology, Begumpet, Hyderabad.

Books by Sir Ronald Ross

Ronald Ross was an avid writer who oversaw the publication of multiple books and a large number of scholarly articles. Table 1 shows some of his well-known works of writing and publications.

**Table 1:** Ronald Ross’s well-known works of writing and publications.

Category	Title	Year
Scientific work	Mosquito Brigades and How to Organize Them [18]	1902
	The Prevention of Malaria [19]	1910
Literacy & fiction work	Memoirs, With a Full Account of the Great Malaria Problem and its Solution [20]	1923
	Lyra Modulata [21]	1931
	Fables and Satires [22]	1903
Selected papers	On Some Peculiar Pigmented Cells Found in Two Mosquitoes Fed on Malarial Blood [23]	1897
	The role of the mosquito in the evolution of the malarial parasite: the recent researches of Surgeon-Major Ronald Ross, I.M.S. 1898 [24]	1898

## Conclusions

Sir Ronald Ross’s ground-breaking findings not only improved our understanding of malaria but also had a significant impact on public health approaches to the disease, saving many lives and providing the groundwork for future research and control initiatives. Despite enormous challenges, Ross's dedication to scientific inquiry led to his rich legacy to the world. Beyond his scientific achievements, he was a multitalented personality, contributing to literature, poetry, and music.
